# Small Molecule “Silmitasertib” Repurposed as Inhibitor of Transforming Growth Factor Beta 1 for the Development of Therapeutics for Oral Submucous Fibrosis

**DOI:** 10.1155/2021/6631848

**Published:** 2021-04-01

**Authors:** Nezar Boreak

**Affiliations:** Department of Restorative Dental Sciences, College of Dentistry, Jazan University, Jazan, Saudi Arabia

## Abstract

Oral submucous fibrosis (OSMF) is considered a premalignant condition characterized by aggressive fibrosis of the submucosal tissues of the oral cavity reflecting its malignant transformation potential. Activation of transforming growth factor beta (TGF-*β*) signaling has been reported to lead increased collagen production and fibrosis. Recently, significant upregulation of TGF-*β*1 has been reported in OSMF as compared to normal tissues. Therefore, inhibition of the TGF-*β*1 may pave for the development of therapeutics of OSMF. Based on the structure-assisted drug designing, we found “silmitasertib” as potent inhibitor of TGF-*β*1. We suggest that this molecule can be validated and implemented for the treatment of OSMF.

## 1. Introduction

Oral submucous fibrosis (OSMF) is considered a premalignant condition characterized by aggressive fibrosis of the submucosal tissues of the oral cavity reflecting its malignant transformation potential [1]. Submucosal fibrosis usually affects oral cavity, pharynx, and esophagus leading to dysphagia and progressive trismus and increases the risk for development of cancer [2]. Activation of transforming growth factor beta (TGF-*β*) signaling has been reported to lead increased collagen production, and fibrosis and significant upregulation of TGF-*β*1 have been reported in OSMF as compared to normal tissues [3]. Areca nut chewing has been reported the most probable cause of OSMF [3–5]. Epidemiological evidences reflect exponential increase in OSMF in younger male population [6]. Studies indicated that near about 10% OSMF cases are associated with malignant transformation to oral squamous cell carcinoma (OSCC) [7, 8].

Many treatment modalities for OSMF have been proposed, but none has shown any significant effect so far [9]. Therefore, the search for effective anti-OSMF agents still continues. It has been reported that areca nut chewing regulates TGF-*β*1 signaling in epithelial cells, which in turn affects the nearby fibroblasts leading canonical downstream SMAD signaling activation that results in mesenchymal interaction leading to fibrosis [10–14]. Due to functional importance of this TGF-*β*1 signaling, TGF-*β*1 is proposed as a potent therapeutic target for the development of anti-OSMF drugs. To facilitate the targeted therapy for OSMF, we carried out the structure-assisted screening of existing small molecules to target TGF-*β*1 [15]. Here, we report a small molecule “silmitasertib” as a potent inhibitor of TGF-*β*1. Findings support the premise that this promising small molecule can be validated and implemented for the treatment of OSMF.

## 2. Material and Methods

### 2.1. Target Preparation and Small Drug Molecules

The crystal structure of target protein, TGF-*β*1, was downloaded from Protein Data Bank (PDB ID: 1KLA), and energy minimization was carried out using SPDBV [16]. Structure was explored for different potential problems such as missing atoms, alternate locations, more than one molecule, added waters, and chain breaks. Polar hydrogen's were added, and the Kollman United Atom Charges were assigned. Chemical structures of all small molecules (*n* = 1137) were downloaded from Drug Bank and PubChem and processed in ChemBioDraw® Ultra 12.0 [17].

### 2.2. Molecular Docking and MD Simulations

Molecular docking and MD simulation studies were carried out on DELL® workstation with Intel® Xeon® CPU E5-2609 v3@1.90 GHz processor, 64 GB RAM, and two-terabyte hard disk running on Ubuntu 14.04.5 LTS operating system. Docking was carried out using AutoDock Vina [18]. GROMACS 5.1.1 utilities were used for MD simulations. Computational tools such as PyMOL, Discovery Studio Visualizer, and QtGrace [18, 19] were used for visualization, evaluation, and analysis of MD trajectories.

MD simulations (100 ns) of TGF-*β*1 and TGF-*β*1-silmitasertib complex were carried out at 300 K at the molecular mechanics level using GROMOS96 43A1 force-field in GROMACS 5.1.1. Conformations were sampled at every 10 ps during 100 ns simulations of TGF-*β*1 and the TGF-*β*1-silmitasertib complex. Resulting trajectories were analyzed by using GROMACS 5.1.1 utilities namely *gmx energy*, *gmx rms*, *gmxrmsf*, *gmx gyrate*, *gmx sham*, and *gmx sasa*. All the graphs and figures were plotted using QtGrace [19].

### 2.3. Calculation of Inhibition Constant

Inhibition constant (*Ki*; nM) was calculated from the Δ*G* parameter using the formula

(1) $$\begin{array}{c} Ki=\text{EXP}\left(\left(\Delta G*1000\right)/\left(\text{R}*\text{T}\right)\right), \end{array}$$

where Δ*G* is the docking energy, *R* = 1.98719 cal K^−1^ mol^−1^, and *T* = 298.15°K:

(2) $$\begin{array}{c} Ki=\text{EXP}\left(\left(A*1000\right)/\left(198719*29815\right)\right). \end{array}$$

## 3. Results

### 3.1. Interaction Analysis of TGF-*β*1 with Small Molecules

Molecular docking analysis was carried out to explore the binding energy, binding affinity, and bound conformations of potential interacting amino acid residues along with their intermolecular distances. All small molecules (*n* = 1137) were blindly docked against the target molecule TGF-*β*1. Top five molecules along with binding energy and inhibition constant are represented in Table 1. Based on the high-binding energy values and significant amino acid residual interactions, we report “silmitasertib” as potent inhibitor of TGF-*β*1 (Tables 1 and 2).

**Figure 1:**
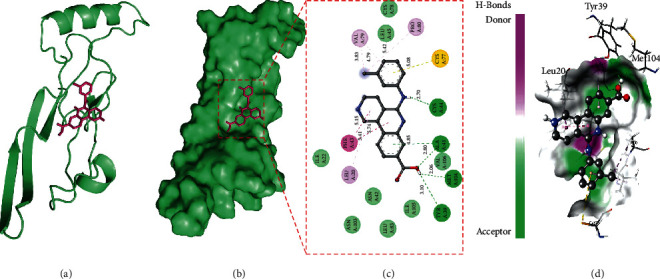
The crystal structure of TGF-*β*1 in complex with silmitasertib. (a) A cartoon representation of the TGF-*β*1-silmitasertib complex. (b) Surface representation of TGF-*β*1. Silmitasertib is represented in red-colored sticks. (c) A zoomed view of substrate-binding pocket representing the key amino acid residues forming interactions with inhibitor molecule. (d) Surface representation of conserved substrate-binding pocket of TGF-*β*1.

Here, we present TGF-*β*1 in complex with silmitasertib (Figure 1(a)). This complex showed that silmitasertib binds on the substrate-binding site present between domains I and II of TGF-*β*1. Binding of inhibitor with in this cleft offers that this interaction may decrease substrate accessibility to TGF-*β*1 and therefore be responsible for its inhibition. Silmitasertib was subjected to further molecular docking analysis to explore best docking pose on the entire target protein surface (Figure 1(b)), reflecting a strapping binding pattern of silmitasertib within the main groove of TGF-*β*1.

Next, the TGF-*β*1-silmitasertib complex was analyzed to reveal the bonding pattern of potent amino acid residues. Results showed that TGF-*β*1 offered many potential hydrogen bonds with reliable bond distance to silmitasertib (Table 2 and Figures 1(c) and 1(d)). Bond distances are represented in Figure 1(c). Results showed that silmitasertib formed major interactions with TYR39, ALA41, CYS44, and MET104. The geometric properties of these hydrogen bonds revealed that they surrounded the active site of TGF-*β*1. In addition to these major interactions, TGF-*β*1 also formed other types of interactions such as Pi-Pi T-shaped, Pi-sulfur, alkyl, Pi-alkyl, and Van der Waals with silmitasertib (Table 2 and Figures 1(c) and 1(d)).

### 3.2. Dynamics of the TGF-*β*1-Silmitasertib Complex

Molecular dynamics simulations (100 ns) of free TGF-*β*1 and the TGF-*β*1-silmitasertib complex were carried out to analyze the conformational changes, interaction, and stability. Prior to MD analysis, the average potential energy of both free TGF-*β*1 and the TGF-*β*1-silmitasertib complex was determined. The average potential energy of -888944 kJ mol^−1^ and -556290 kJ mol^−1^ for TGF-*β*1 and the TGF-*β*1-silmitasertib complex was observed, respectively, reflecting the stability and equilibration of systems. After completion of the simulation process, we calculated the root-mean square deviation (RMSD), root-mean square fluctuation (RMSF), radius of gyration (*Rg*), solvent accessible surface area (SASA), kinetic energy, enthalpy, volume, and density of the systems. Values of all these parameters are represented in Table 3.

**Figure 2:**
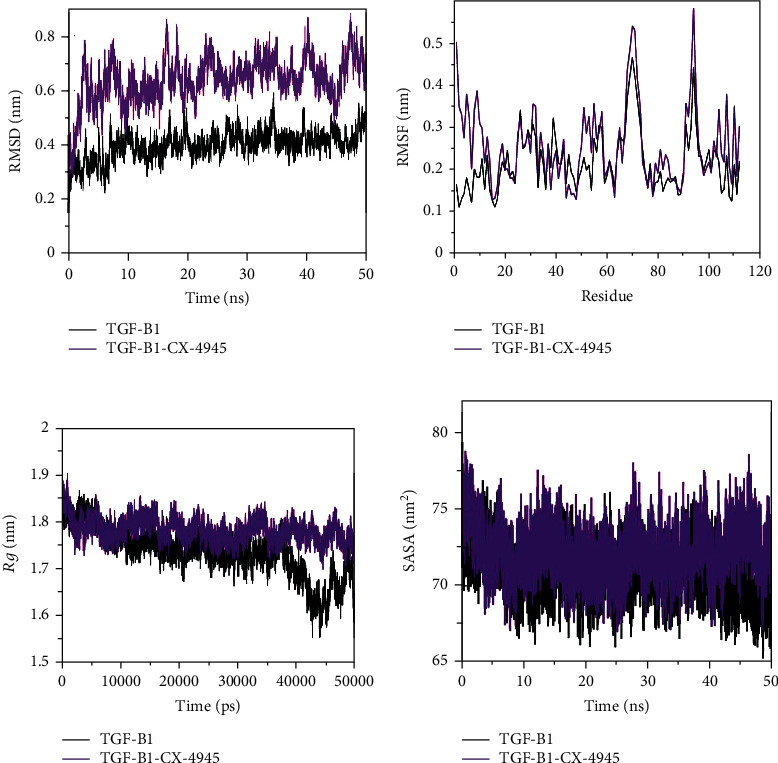
Structural dynamics of TGF-*β*1 up on silmitasertib binding. (a) The RMSD plot of TGF-*β*1 as a function of time. (b) RMSF plot of free TGF-*β*1 and upon silmitasertib binding. (c) Time evolution of radius of gyration. (d) The SASA plot of TGF-*β*1 as a function of time. The values were obtained from 100 ns MD simulations time scale. Black and blue represent values obtained for free TGF-*β*1 and the TGF-*β*1-silmitasertib complex, respectively.

Attachment of ligands in the binding pocket of target protein can lead to structural deviations and conformational changes and can alter its stability [20]. These structural and conformational deviations and stability can be evaluated by calculating the RMSD [21]. We observed the RMSD values of 0.399029 nm and 0.638279 nm for TGF-*β*1 and TGF-*β*1-silmitasertib, respectively (Table 3). The RMSD plot (Figure 2(a)) showed that attachment of silmitasertib in the binding pocket of TGF-*β*1 led minimal structural and conformational deviations in the native structure of TGF-*β*1. RMSD also showed that there are some random fluctuations. These fluctuations occur due to initial orientation of silmitasertib in the binding pocket of TGF-*β*1. However, thereafter, the system attained stable equilibrium throughout the simulation. These findings reflect that binding of silmitasertib to TGF-*β*1 does not alter the stability and structure of TGF-*β*1. Further, least RMSD at several parts and equilibration throughout the simulation strongly suggests the stability of the TGF-*β*1-silmitasertib complex (Figure 2(a)).

In order to perceive the local structure flexibility, RMSF of both TGF-*β*1 and TGF-*β*1-silmitasertib complex was calculated and plotted (Figure 2(b) and Table 3). RMSF represents the average fluctuation of all residues. RMSF showed many residual fluctuations at distinct regions in the TGF-*β*1 structure, which were minimized upon silmitasertib binding during the simulation process. However, several random residual fluctuations were also observed upon silmitasertib binding with TGF-*β*1 (Figure 2(b)).

We computed the *Rg*, which is directly related to the overall conformational shape and tertiary structure volume of a protein, reflecting the stability of the protein in a biological system. A protein is supposed to have a higher *Rg* due to flexible packing. The average *Rg* values, 1.73424 nm and 1.78123 nm, were observed for TGF-*β*1 and the TGF-*β*1-silmitasertib complex, respectively (Table 3). No substantial deviations were observed in the packing of TGF-*β*1 in presence of silmitasertib as a complex ensemble in the *Rg* plot. The *Rg* plot indicated initial higher compactness up to 40 ns of MD trajectory, which the protein subsequently attained. The *Rg* plot indicates initial higher compactness up to 40 ns of MD trajectory, which may be due to tight protein packaging; but the protein subsequently achieved stable *Rg* equilibrium in the simulation (Figure 2(c)). The *Rg* plot showed minimum structural deviation and no TGF-*β*1 conformation change to silmitasertib binding. The *Rg* plot revealed that even after silmitasertib binding, TGF-*β*1 remained tightly packed throughout the simulation.

Next, we calculated the SASA 100 ns MD simulations, representing the surface area of a protein interacting with surrounding solvent [22]. SASA is directly related to the *Rg* of a protein. The average SASA values, 70.8811 nm^2^ and 72.301 nm^2^, were observed for TGF-*β*1 and the TGF-*β*1-silmitasertib complex, respectively (Figure 2(d)). Increment in SASA in the case of the TGF-*β*1-silmitasertib complex is presumed due to conformational change that renders the exposure of some of internal residues in TGF-*β*1 to solvent.

### 3.3. Dynamics of Interactions in the TGF-*β*1-Silmitasertib Complex

**Figure 3:**
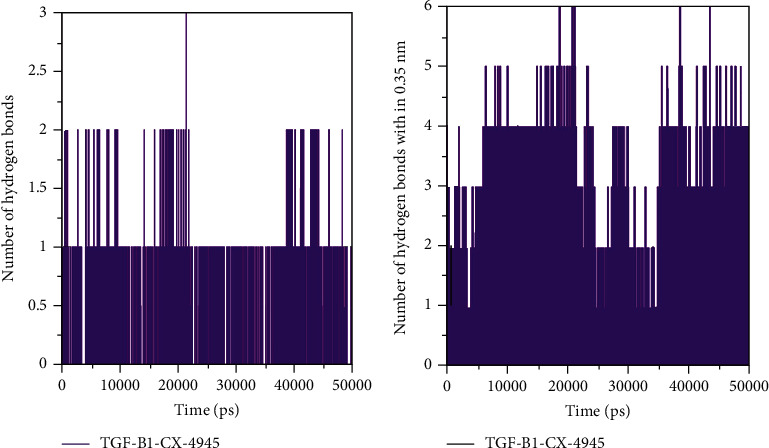
Time evolution and stability of hydrogen bonds formed. (a) Hydrogen bonds between TGF-*β*1 and silmitasertib. (b) Hydrogen bonds paired within 0.35 nm between TGF-*β*1 and silmitasertib.

A basic feature of protein stability is its intramolecular bonding. The H-bonds can be used to determine the stability of polar interactions between a protein and a ligand, providing directionality and interaction specificity reflecting a fundamental feature of molecular recognition. To evaluate and validate the stability of TGF-*β*1 and the TGF-*β*1-silmitasertib complex, we calculated the H-bonds along with the bonds paired within 0.35 nm during the simulation. The average number of H-bonds in TGF-*β*1 after silmitasertib binding was found 0.149 (Figures 3(a) and 3(b)). H-Bond's analysis showed that silmitasertib binds on the active site of TGF-*β*1 with least fluctuations.

### 3.4. Secondary Structure Cha4nges in the TGF-*β*1-Silmitasertib Complex

**Figure 4:**
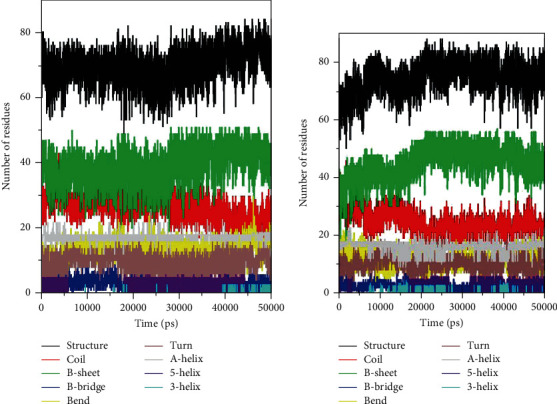
Secondary structure content of (a) free TGF-*β*1 and (b) TGF-*β*1-silmitasertib complex. ∗Structure = *α* − helix + *β* − sheet + *β* − bridge + turn.

We evaluated the secondary structural components of TGF-*β*1 in order to determine the overall structural changes in TGF-*β*1 upon silmitasertib binding as a function of time. For each time step, secondary structure components (a-helix, b-sheet, and turns) of TGF-*β*1 were bust into individual residues, and their average number in structure formation was plotted as a function of time. We observed no change in the structural components of free TGF-*β*1 throughout the simulation. All the structural elements remained constant and equilibrated (Figure 4(a)). However, little changes were observed in structure, coil, and B-sheet in TGF-*β*1 binding of silmitasertib (Figure 4(b)). The average number of residues participated in secondary structure formation in the case of the TGF-*β*1-silmitasertib complex was found to be slightly changed due to coil and bend formation as compared with free TGF-*β*1 (Figure 4(b) and Table 4). Here, no major changes were seen in the secondary structure content of TGF-*β*1 upon silmitasertib binding which further supports a strong stability of the complex.

## 4. Discussion

Oral submucous fibrosis (OSMF) is a chronic devastating disease of the oral cavity and is considered a premalignant condition [1]. Pathological characteristics include chronic inflammation, excessive collagen deposition in the connective tissues below the oral mucosal epithelium, local inflammation in the lamina propria or deep connective tissues, and degenerative changes in the muscles [23]. In OSMF, aggressive fibrosis of the submucosal tissues increases the risk for development of cancer [2]. Significant upregulation of TGF-*β*1 has been reported in OSMF [3]. Emerging studies are reflecting exponential increase in OSMF in younger male population [6, 23]. Related literature indicates that about 10-15% OSMF cases were found associated with malignant transformation [7, 8, 23]. There are many treatment modalities that have been previously implemented for the treatment of OSMF, but none has shown any significant effect so far [9]. Therefore, the search for effective anti-OSMF agents still continues. It has been reported that upregulated TGF-*β*1 signaling in epithelial cells affects the nearby fibroblasts leading canonical downstream SMAD signaling activation that results in mesenchymal interaction leading to fibrosis [10–14]. Due to functional importance of this TGF-*β*1 signaling, TGF-*β*1 is proposed as a potent therapeutic target for the development of anti-OSMF drugs.

Computational drug discovery is considered an effective strategy for accelerating drug discovery [24–26]. The applicability of computational drug discovery has been broadly applied to nearly every stage in the drug discovery and development including target identification and validation, lead discovery and optimization, and preclinical tests [24, 26]. Based on the large-scale availability of small molecules and biological macromolecules, structure-assisted screening methods are most commonly implemented for the discovery of small molecules bearing drug-like properties [24].

In the current study, molecular docking analysis was carried out to explore the binding energy, binding affinity, and bound conformations of potential interacting amino acid residues along with their intermolecular distances [18, 19]. Based on the high-binding energy value (-9.3 kcal/mol) and significant amino acid residual interactions, we report “silmitasertib” as a potent inhibitor of TGF-*β*1. The best docking pose on the entire target protein surface reflected a strapping binding pattern of silmitasertib within the main groove of TGF-*β*1. TGF-*β*1 offered many potential hydrogen bonds to silmitasertib through TYR39, ALA41, CYS44, and MET104. Hydrogen bonds play a crucial role in determining the specificity of ligand binding [27]. TGF-*β*1 also formed other types of interactions such as Pi-Pi T-shaped, Pi-sulfur, alkyl, Pi-alkyl, and Van der Waals with silmitasertib. All these bonding interactions collectively contribute for the stability of the protein-inhibitor complex [27]. Identification of silmitasertib was followed by MD simulations (100 ns) of free TGF-*β*1 and the TGF-*β*1-silmitasertib complex to analyze the conformational changes, interaction, and stability. MD simulations have evolved into a mature technique that can be used effectively to understand macromolecular structure-to-function relationships [28]. Prior to MD analysis, the average potential energy of TGF-*β*1 (-888944 kJ mol^−1^) and the TGF-*β*1-silmitasertib complex (-556290 kJ mol^−1^) reflected the stability and equilibration of systems. Characterization of four MD parameters (RMSD, RMSF, *Rg*, and SASA) reflected silmitasertib as a potent inhibitor of TGF-*β*1. It is well understood that binding of ligand produces structural deviations and conformational changes, and can alter stability of the target macromolecule [20]. These structural and conformational deviations and stability can be evaluated by calculating the RMSD [21]. Comparative RMSD values showed that silmitasertib binding with TGF-*β*1 led minimal structural and conformational deviations in the native structure of TGF-*β*1, strongly suggesting the stability of the TGF-*β*1-silmitasertib complex. RMSF presents local structure flexibility [21]. Initially, we found many residual fluctuations at distinct regions in the TGF-*β*1 structure, which were minimized upon silmitasertib binding during the simulation process, reflecting the structure flexibility TGF-*β*1-silmitasertib complex. *Rg* is directly related to overall conformational shape and tertiary structure volume of a protein, reflecting stability of the protein in a biological system [21]. The *Rg* plot showed minimum structural deviation and no TGF-*β*1 conformation change to silmitasertib binding. The *Rg* plot revealed that even after silmitasertib binding, TGF-*β*1 remained tightly packed throughout the simulation. SASA presents the surface area of a protein interacting with surrounding solvent [22]. We found increment in SASA in the case of the TGF-*β*1-silmitasertib complex that is presumed due to conformational change that renders the exposure of some of internal residues in TGF-*β*1 to solvent. Molecular screening along with RMSD, RMSF, *Rg*, and SASA computations confirms the strong and stable binding of silmitasertib with TGF-*β*1. A basic feature of protein stability is its intramolecular bonding. The H-bonds can be used to determine the stability of polar interactions between a protein and a ligand, providing directionality and interaction specificity reflecting a fundamental feature of molecular recognition [28]. The average number of H-bonds in TGF-*β*1 after silmitasertib binding was found 0.149, reflecting binding of silmitasertib on the active site of TGF-*β*1 with least fluctuations. Moreover, we analyzed secondary structure changes in the TGF-*β*1-silmitasertib complex to determine the overall structural changes in a complex function of time [18, 19]. We observed that the structural elements remained constant and equilibrated in TGF-*β*1, while little changes were observed in structure, coil, and B-sheet in the TGF-*β*1-silmitasertib complex. The average number of residues that participated in the secondary structure formation in the case of the complex was found to be slightly changed due to coil and bend formation. Overall, no major changes were seen in the secondary structure content of TGF-*β*1 upon silmitasertib binding which further supports a strong stability of the complex.

## 5. Conclusion

Oral submucous fibrosis (OSMF) is considered a premalignant condition characterized by aggressive fibrosis of the submucosal tissues of the oral cavity reflecting its malignant transformation potential. Activation of transforming growth factor beta (TGF-*β*) signaling has been reported to lead increased collagen production and fibrosis. Recently, significant upregulation of TGF-*β*1 has been reported in OSMF as compared to normal tissues. Therefore, inhibition of the TGF-*β*1 may pave for the development of therapeutics of OSMF. Based on the structure-assisted drug designing approach, we conducted the screening of 1137 small molecules. Molecular docking and simulation analysis revealed a small molecule “silmitasertib” as a potent inhibitor of TGF-*β*1. Findings support the premise that this promising small molecule can be validated and implemented for the treatment of OSMF.

## Figures and Tables

**Table 1:** Binding parameters of selected small drug molecules with TGF-*β*1.

S. No.	Small drug molecules	Target protein	Binding affinity (kcal/mol)	Inhibition constant, *Ki* (nM)
1	Silmitasertib	TGF-*β*1	-9.3	1.61889E-06
2	SR10067		-7.8	1.91655E-06
3	Neoruscogenin		-7.5	3.17997E-06
4	SR1078		-7.3	4.45681E-06
5	SRT2183		-7.3	4.45681E-06

**Table 2:** Interacting amino acid residues of top-five candidate small drug molecules proposed as potent inhibitors of TGF-*β*1.

S. No.	Target protein	Small drug molecules	Interaction type	Interacting amino acid residues
1	TGF-*β*1	Silmitasertib	Hydrogen bond	TYR39, ALA41, CYS44, and MET104
			Pi-pi T-shaped, pi-sulfur	PHE43, CYS77
			Alkyl, pi-alkyl	LEU20, VAL79, and PRO80
			Van der Waals	ILE22, ANS42, LEU45, CYS78, LEU83, ASN103, ILE105, and VAL106
2		SR10067	Hydrogen bond	CYS44
			Alkyl, pi-alkyl	TRP30, PRO80, and LEU83
			Pi-pi T-shaped, pi-sulfur	PHE43, MET104, and CYS109
			Van der Waals	LEU20, ILE22, TRP32, TYR39, ALA41, ASN42, VAL79, LEU101, ASN103, and VAL106
3		Neoruscogenin	Hydrogen bond	TYR39, MET104
			Alkyl	ALA41, LEU45, CYS77, VAL79, and PRO80
			Van der Waals	LEU20, ILE22, ASN42, PHE43, CYS44, GLY46, and CYS78
4		SR1078	Hydrogen bond	ARG25, LYS31, HIS34, and ARG94
			Pi-alkyl, pi-pi stacked, pi-pi T-shaped	HIS34, TYR91
			Van der Waals	PHE24, TRP32, and GLY93
5		SRT2183	Hydrogen bond	LY31, GLY93
			Pi-alkyl, pi-pi stacked	ARG25, LYS31, and TYR91
			Van der Waals	TRP30, ILE33, HIS34, VAL92, and ARG94

**Table 3:** Calculated MD parameters for the TGF-*β*1 and TGF-*β*1-silmitasertib systems obtained after simulation.

Complex	Average RMSD (nm)	Average RMSF (nm)	Average *Rg* (nm)	Average SASA (nm^2^)	Kinetic energy	Enthalpy	Volume (nm3)	Density (g 1-1)
TGF-*β*1	0.399029	0.218281	1.73424	70.8811	142987	-745922	576.511	1009
TGF-*β*1-silmitasertib	0.638279	0.257439	1.78123	72.301	102625	-453640	422.08	984.903

**Table 4:** Percentage of residues participated in average structure formation.

Complex	Percentage of protein secondary structure								
	Structure	Coil	*β*-Sheet	*β*-Bridge	Bend	Turn	*α*-Helix	5-Helix	3-Helix
TGF-*β*1	0.62	0.24	0.35	0.03	0.12	0.08	0.16	0.02	0.00
TGF-*β*1-silmitasertib	0.67	0.21	0.41	0.02	0.10	0.10	0.15	0.01	0.00

## Data Availability

All the data has been included in the manuscript.
